# Pragmatic randomized controlled trial comparing a complex telemedicine-based intervention with usual care in patients with chronic conditions

**DOI:** 10.1007/s10198-023-01664-w

**Published:** 2024-01-31

**Authors:** Susanna Sten-Gahmberg, Kine Pedersen, Ingrid Gaarder Harsheim, Hanna Isabel Løyland, Øyvind Snilsberg, Tor Iversen, Geir Godager, Erik Magnus Sæther, Birgit Abelsen

**Affiliations:** 1 Oslo Economics, Klingenberggata 7, 0161 Oslo, Norway; 2 Present Address: Department of Health Management and Health Economics, University of Oslo, Postboks 1089 Blindern, 0317 Oslo, Norway; 3 Norwegian Centre for Rural Medicine, Department of Community Medicine, UiT - The Arctic University of Norway, 9037 Tromsø, Norway; 4 Present Address: The Finnish Centre for Pensions, 00065 ELÄKETURVAKESKUS, Finland

**Keywords:** Primary healthcare, Telemedicine, Pragmatic randomized control trial, Effectiveness analysis, Cost–benefit analysis, Chronic disease, I18

## Abstract

This study evaluates a complex telemedicine-based intervention targeting patients with chronic health problems. Computer tablets and home telemonitoring devices are used by patients to report point-of-care measurements, e.g., blood pressure, blood glucose or oxygen saturation, and to answer health-related questions at a follow-up center. We designed a pragmatic randomized controlled trial to compare the telemedicine-based intervention with usual care in six local centers in Norway. The study outcomes included health-related quality of life (HRQoL) based on the EuroQol questionnaire (EQ-5D-5L), patient experiences, and utilization of healthcare. We also conducted a cost–benefit analysis to inform policy implementation, as well as a process evaluation (reported elsewhere). We used mixed methods to analyze data collected during the trial (health data, survey data and interviews with patients and health personnel) as well as data from national health registers. 735 patients were included during the period from February 2019 to June 2020. One year after inclusion, the effects on the use of healthcare services were mixed. The proportion of patients receiving home-based care services declined, but the number of GP contacts increased in the intervention group compared to the control group. Participants in the intervention group experienced improved HRQoL compared to the control group and were more satisfied with the follow-up of their health. The cost–benefit of the intervention depends largely on the design of the service and the value society places on improved safety and self-efficacy.

## Background

Healthcare systems in industrialized countries, including Norway, face challenges due to aging populations and an increased burden of chronic conditions. These factors combined increase both the need for and the complexity of healthcare service provision [1]. Residents with chronic conditions demand interdisciplinary and comprehensive follow-up over time, requiring sufficient capacity and competence in the healthcare system. There is a need for the development of efficient and cost-effective services that can adequately follow-up individuals.

Telemedicine has been proposed as a tool to improve the efficiency of healthcare because it allows sharing and coordinating resources that are geographically distant [2]. Using digital technology, patients can share health information with a care provider in real-time from their homes instead of physical face-to-face interactions. In accordance with technology advancements, the use of telemedicine has increased significantly over time [3]. Leonardsen and co-workers [4] summarized empirical studies exploring patient experiences with telemedicine interventions, finding that patients feel more empowered, they learn more about their condition (health competence), they increase their awareness of symptoms and treatment, and feel safer and more self-efficient. Thus, telemedicine can move healthcare services towards a greater degree of self-efficacy and independence through patient involvement. However, technology barriers, lack of computer literacy, lack of financial incentives, human inertia, organizational and culture issues in healthcare organizations pose barriers to widespread use [5–9].

Although a wealth of telemedicine research exists, individual studies and meta-analyses vary considerably in terms of the type of technology and patients under study. This limits general conclusions about the effectiveness, efficiency, and feasibility of implementation within the healthcare system. While some interventions seem to be effective in one clinical context, they provide little or no benefit in others [10]. For example, one meta-analysis found that telemedicine interventions can significantly lower HbA1c values among patients with diabetes, while other studies reported no or a negative effect [11]. Telemedicine interventions were also found to reduce COPD exacerbations in some, but not all, studies included in a review [12]. Furthermore, telemedicine studies vary with respect to the type of involved personnel, and some controlled studies lack information about patients in control groups [13]. As primary healthcare services vary with respect to content, organization and quality across countries, the generalizability of studies may be limited. Flumignan and co-workers [14] state that there is still insufficient evidence to determine what types of telemedicine interventions are effective, for which patients and in which settings, and whether such interventions can be used as a replacement for standard physical face-to-face treatment. They advocate more use of randomized trials to increase the level of evidence and to reduce potential bias and confounding.

Randomized trials can be classified as either explanatory or pragmatic [15]. Explanatory trials aim at both estimating efficacy and understanding the biological underpinnings of differences between treatments. They tend to include highly selected patients and follow a strict treatment protocol, which limits the generalizability of findings to real-life settings. Pragmatic randomized trials, on the other hand, offer opportunities to combine the real-life complex nature of an observational study with the scientific rigor of a randomized controlled trial, thus improving the scientific quality while being relevant to inform current practice [16]. At the same time, ‘usual care’ is the preferred comparator in pragmatic trials [15], which may vary substantially across service providers and thus increase the complexity in interpreting results.

Pragmatic randomized trials are by nature complex interventions. Accompanying research should account for the complexity that arises from the interventions’ components and from their interaction with the context in which it is being implemented. A new framework for developing and evaluating complex interventions shifts the binary focus of effectiveness to whether and how the intervention will be acceptable, implementable, cost-effective, scalable, and transferable across contexts [17]. The framework identifies four research phases: development or identification of the intervention, feasibility, evaluation, and implementation. Each phase has a common set of core elements—considering context, developing, and refining program theory, engaging stakeholders, identifying key uncertainties, refining the intervention and economic considerations.

This study reports on a pragmatic randomized controlled trial. The aim was to explore the use of a complex telemedicine-based intervention in the follow-up of patients with chronic health conditions within a primary care setting in Norway, in terms of clinical effectiveness, resource use, and real-life implementation challenges. The study seeks answers to the following questions:

- Does the telemedicine-based intervention provide better health than usual care?
- Does the telemedicine-based intervention provide a better patient experience than usual care?
- Does the telemedicine-based intervention imply lower costs of healthcare services than usual care?
- Is the telemedicine-based intervention cost-effective?

The intervention is considered complex due to the heterogeneity of patients targeted and the level of flexibility in the components of the intervention. Our research approach was inspired by the framework for evaluating complex interventions [15].

This study has several contributions to the literature. First, we provide new evidence of the effectiveness of a telemedicine-based intervention in a primary healthcare setting, in terms of both patient health, patient experience and healthcare utilization. Second, we provide a cost–benefit analysis of the intervention which can guide decision-making in the field. Third, in combination with the results presented in Abelsen et al. [18] and Sten-Gahmberg et al. [19], this study has several important methodological contributions. We demonstrate how a complex intervention can be implemented in a real-life setting through a pragmatic randomized control trial. We also display the value and importance of using a mixed-methods approach in the evaluation of such a complex intervention. Thus, this study can lead the way for future research in the field.

## Methods

### Context

The trial was designed as a pragmatic, non-blinded, multi-center, individual, randomized-controlled trial at six centers of varying patient numbers in Norway [18]. These centers were situated in and run by six Norwegian municipalities. A complex but structured telemedicine-based intervention (described below) was compared to usual care in follow-up of patients with chronic conditions. The CONSORT 2010 Checklist was used when developing this manuscript [20].

The trial was carried out in line with the guidelines of the Norwegian Directorate of Health (NDH), who was the principal for the trial. The purpose of the trial was to obtain knowledge about the consequences of telemedicine-based patient follow-up compared to usual primary care follow-up for patients with chronic health conditions. It was important for the NDH to allow service development during the study period, to inform future service design and implementation. This aspect led to variations in the composition of the telemedicine intervention and in the study population, both across the centers and within each center over time.

**Fig. 1:**
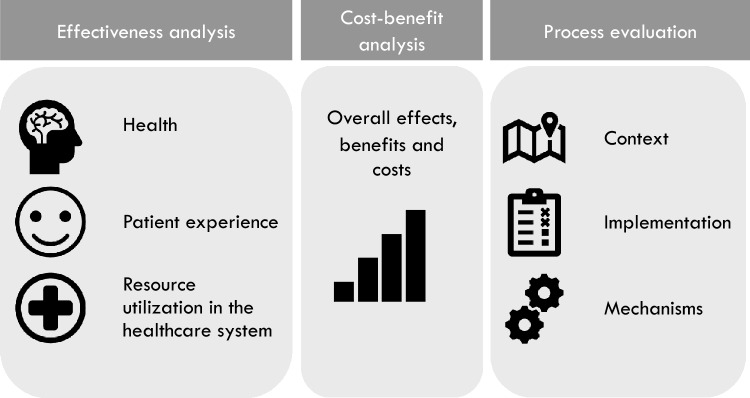
Analytic components of the trial

The outcomes were assessed with mixed methods, with three main analyses: 1) an effectiveness analysis aimed at measuring outcomes in terms of patients' health status, user experience and resource utilization in the healthcare system; 2) a process evaluation aimed at studying aspects of context, implementation, and mechanisms of impact; and 3) a cost–benefit analysis aimed at evaluating the societal value of the telemedicine-based follow-up compared to usual care (Fig. 1). The results from the effectiveness and the cost–benefit analyses are presented in this article, while the results from the process evaluation are presented elsewhere [18, 19].

### Randomization

Patients were randomized to either telemedicine-based follow-up (the intervention group) or usual care (the control group) in a 1:1 relationship. The randomization was carried out using sealed envelopes containing a paper sheet stating either “intervention group” or “control group”. The centers received envelopes grouped in bunches of six (including three sheets with “intervention group” and three sheets with “control group”). The centers assigned one bunch of envelopes to each GP involved in the trial. When a patient was enrolled in the study, the patient and a nurse at the center would draw one envelope from the bunch and open it jointly. If a single GP had more than six participating patients, a new bunch of six envelopes was assigned to that GP.

Inclusion through randomization of patients ended on March 16th, 2020. As patients in the target population were at an elevated risk if infected with COVID-19, it was decided to minimize physical meetings related to inclusion in the study. From March 17th through June 30th, 2020, all eligible patients were included in the study in a non-randomized intervention group. In this study, the randomized and non-randomized intervention groups are treated as one group.

### Trial period

Patients were recruited to the trial from February 19th, 2019, through June 30th, 2020. Data were collected during the period January 1st, 2017, through June 30th, 2021. The follow-up period for participants was 12–18 months depending on the date of inclusion.

### Participant inclusion criteria

Both health personnel and patients themselves could suggest patients for inclusion in the trial. Inclusion criteria were:

-  ≥ 18 years of age
- Considerable disease burden and comprehensive medical needs as judged by health personnel
- At least one chronic condition
- Medium to high risk of worsening of health condition, hospitalization, or increased need for medical and care services
- High consumption of healthcare services
- Reduced level of physical functioning as judged by health personnel
- Motivation and potential to benefit from telemedicine follow-up

Patients without the capacity to consent, substance abusers, and those unable to handle the tablet and the measuring equipment were excluded.

The follow-up centers evaluated compliance with the inclusion criteria. The patient's GP made the final assessment of whether the patient should be included in the trial.

Eligible patients were invited to a meeting, typically a home visit, where they were given written and oral information about the trial and about the intervention. The patients agreed to participate by signing a consent form.

### The telemedicine-based intervention

#### Organization and responsibilities

**Fig. 2:**
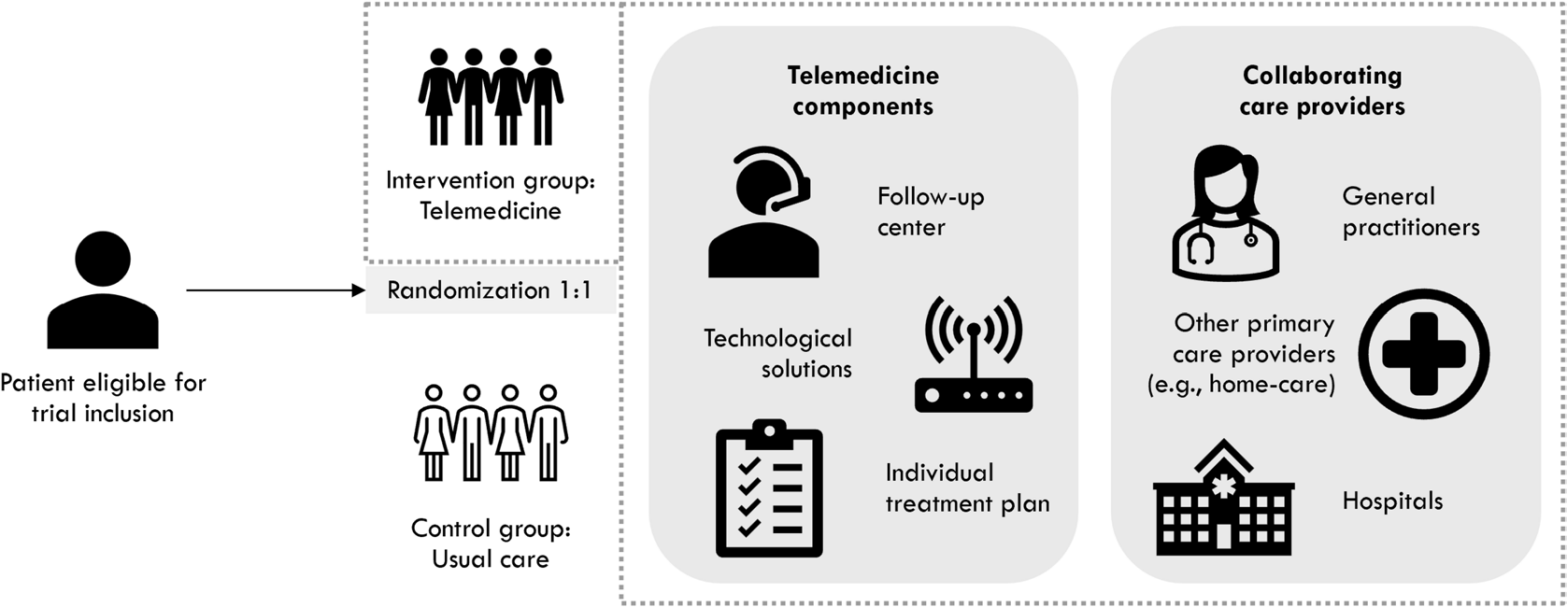
Overview of the complex telemedicine-based intervention

The NDH outlined guidelines for the organization of the telemedicine-based intervention, yet the six centers could adjust the intervention according to local context and needs (Fig. 2). The centers were responsible for recruiting patients to the study, providing the intervention, and facilitating cooperation between GPs, the follow-up center, other primary health and care services and hospitals. The centers were also responsible for technical equipment and software, including procurement, logistics, training in the use of equipment and user support. Three different suppliers of technological solutions were involved.

In addition, the centers differed with respect to their target population (main diagnoses and disease burden), recruitment channels, and the design of the intervention itself.

#### Intervention group follow-up

Once a participant was randomized to telemedicine-based follow-up, the patient received a computer tablet and relevant home telemonitoring devices and was offered user training. The tablet was used to answer simple questions about the patient’s own health and/or to monitor measurements related to health status (such as blood pressure, blood sugar, oxygen saturation or weight). The measurements were automatically transferred from the home telemonitoring devices to the patient’s tablet and to the follow-up center.

In the first few weeks after inclusion, the follow-up center monitored the participants closely, to get to know the patient’s habitual state. After this, a follow-up center nurse, the patient, and their GP prepared an individual treatment plan based on the patient's goals, disease burden and risk of deterioration. The treatment plan was based on a traffic light model, outlining different medicinal and non-medicinal actions that the patient could take depending on whether his/her measurements were green (normal), yellow or red (slightly or significantly deviating from the habitual state).

Intervention group participants received follow-up from nurses at the follow-up center. The follow-up center and the patient agreed on how often the patient should carry out measurements and answer questions based on the patient’s health condition and diagnoses, as well as individual preferences. Most patients registered measurements daily, but the frequency could also vary over time.

Follow-up center nurses monitored the patient’s measurements and reported health status and provided guidance and medical support based on the patient’s needs. The technological solutions informed the patient and the follow-up center when the measurement results deviated from the patient’s normal values. The threshold values for non-normal measurements were set by the health personnel in consultation with the patient. The nurse responded to abnormal results, and considered, in consultation with the patient, whether the patient should contact the GP or the emergency room. If a patient did not report the scheduled measurements, this could also prompt the follow-up center to contact the patient. Patients could also contact the follow-up center nurse by phone or using the tablet if they had questions regarding their health.

### Control group follow-up

Participants in the control group received usual clinical care. The specific services provided as part of usual clinical care varied according to their health condition and needs. Most patients in the control group were followed up by their GP.

### Analysis and outcome measures

The study involved multiple sources of data, including both quantitative and qualitative data from national registries, study questionnaires and interviews (Table 9 in Appendix). We assessed the effectiveness of the telemedicine intervention compared to usual care, using the following primary outcome measures: 1) change in health-related quality of life (HRQoL), measured using EQ-5D-5L and Visual Analog Scale (VAS) from baseline at 6, 12 and 18 months, 2) change in overall satisfaction with follow-up from baseline at 6, 12 and 18 months (measured through the question “To what extent are you satisfied with the follow-up of your own health?” with answers on a 4-point scale ranging from “Not at all” (1) to “To a large extent” (4)), and 3) change in the use of different types of healthcare services from baseline at 12 months. In addition to the primary outcomes, we assessed secondary outcomes, such as survival, patient’s sense of safety, self-efficacy and understanding of one's own illness.

At inclusion, as well as 6, 12 and 18 months after inclusion, participants were asked about their health-related quality of life (HRQoL), using the standardized EuroQol 5 Dimensions 5 Levels (EQ-5D- 5L) protocol [21] in a questionnaire. In the EQ-5D-5L questionnaire, participants are asked to report their degree of problems with five dimensions of health (mobility, self-care, usual activities, pain/discomfort, and anxiety/depression), where the degree of problems are ranked from level 1 (no problems) to level 5 (extreme problems). The combination of levels within each dimension enables a total of 3125 different health states. Research-based methods have been developed to assign a quality-of-life value between zero and 1.0 to each of the 3125 combinations, where a higher value indicates a better HRQoL [22]. The EQ-5D-5L questionnaire also includes a measure of self-assessed health (Visual Analog Scale – EQ VAS) [23], where respondents assess their own health directly on a scale between 0 (worst possible health imaginable) and 100 (best possible health).

Information about the participants’ use of health and care services was collected from various national registers during the period 2017 to medio 2021 (Table 9 in Appendix). Health and care services include all kinds of services from primary and specialist healthcare providers.

Qualitative data were collected through questionnaires and semi-structured interviews throughout the study period. These data are described in Table 9 in the Appendix but are mainly analyzed in separate work [18, 19].

The statistical method for analyzing the effectiveness data was adapted to the characteristics of the outcome variables in question. For each outcome, we estimated the mean absolute change per patient compared to the baseline value. The null hypothesis was no difference between the intervention and control group. We report whether *p*-values are below 0.1, 0.05 or 0.001. Statistically significant differences between the groups were interpreted as causal effects. To increase the number of observations, we include both the randomized and non-randomized intervention groups. The pragmatic study design created challenges for the population to be used in the effect estimation. An intention to treat (ITT) design estimates the effects for the entire group that is randomized to receive treatment and is often the preferred design from the researcher’s perspective. The present study was closely linked to the policymaker’s perspective. Policymakers were interested in the technology’s potential when service design and patient characteristics match, such that a patient chooses to participate during the entire project period. With information about patient characteristics, service providers can then later select patients who are likely to participate during an entire period. Since the policymaker perspective pulls in the direction of a per-protocol population (PP) design, we use a PP design to analyze the effects of the intervention. The PP population only includes individuals who still received follow-up 12 months after inclusion. Participants who died or ended follow-up for other reasons were excluded. We acknowledge that the PP design reflects the intervention’s efficacy, while the impact policymakers can realistically expect, is reflected by intention to treat. In Table 10 in the Appendix, we show that the two designs give the same results. The table also shows that the results were not affected by the exclusion of the non-randomized arm.

The results concerning differences in development of user experience, HRQoL-measures and healthcare utilization reported here are based on a difference-in-difference (DiD) specification measuring differences in changes in outcomes between baseline and 12 months follow-up. Specifically, we estimate a linear regression model with patient-fixed effects, time-fixed effects and a dummy variable equal to 1 for the intervention group in the study period. The dummy variable coefficient is an estimate of the DiD. Alternatively, we could have used a two-part model where the first part estimates the probability of using a service (extensive margin) and the second part estimates the use of services contingent on positive service utilization (intensive margin). There is a trade-off between less space required to display results from a linear model and the potential ignorance of mass at zero, as for instance with hospital admissions. Analysis of mortality is based on case fatality ratios, and a *t*-test is used to test the difference between groups.

#### Sample size

The NDH decided that 600 participants should be included in the trial. The sample size was decided prior to involvement from the research team, and power calculations were not used in this decision. According to our power calculations, a sample size of 600 is sufficient to detect a difference of 5.5 points on the VAS scale with 80 percent power.

#### Process evaluation

To better understand the local implementation of the intervention and its impact [24], we conducted a process evaluation. In this exploratory analysis, we mainly relied on semi-structured interviews with key actors in the different healthcare contexts and a convenience sample of patients and their next-of-kin (Table 9 in Appendix). The results from this analysis are presented in Sten-Gahmberg et al. [19].

### Cost–benefit analysis

We evaluated the societal value of the telemedicine-based intervention compared to standard clinical care by comparing the total costs and benefits of each treatment arm using cost–benefit analysis. The analysis was based on data from the effectiveness and the process analyses, supplemented with data from national registries, fee schedules, surveys, and interviews with key stakeholders (including qualitative data described in Table 1), and literature review. In accordance with guidelines for conducting cost–benefit analyses from the Norwegian Directorate for Financial Management [25] and guidelines for health economic evaluations from the NDH [26], we included all intended and non-intended effects, both within and outside the healthcare sector.

**Table 1:** Characteristics of participants in the analysis, percentages and averages

	All participants (*N* = 636)	Randomized intervention group (*N* = 230)	Non-randomized intervention group (N = 165)	Randomized control group (*N* = 241)	*N*
Age (mean)	69.7	69.8	68.0	70.9	636
Women	49.0	45.2	53.3	49.6	636
Recruitment diagnosis					636
Diabetes	17.2	19.1	11.5	19.2	
Heart failure	12.1	10.9	10.3	14.6	
COPD	52.0	55.7	50.9	49.2	
Cancer	6.9	5.2	8.5	7.5	
Mental illness	2.8	1.3	8.5	0.4	
Other	7.7	7.8	10.3	9.1	
Health-related quality of life (EQ-5D-5L. mean)	0.613	0.610	0.596	0.628	553
Share with home-based care services	30.9	33.5	31.5	27.9	636
Number of contacts with GP per month	1.73	1.71	1.83	1.68	636
Share with unplanned hospitalization	49.4	45.7	49.1	53.3	636

The use of home-based care services is measured five to eight weeks before inclusion, while the use of GP services and hospital admissions are measured in the last year before inclusion. Source: Participant questionnaire, trial registration form and national registries

We evaluated societal costs and benefits using an analytic time horizon of 12 months, meaning that costs of the intervention reflected follow-up for 12 months from the allocation of the service, while benefits reflected results from the effectiveness analysis using a 12-month time period. This time horizon reflects the follow-up period for most participants.

#### Identification, quantification and valuation of costs and benefits

Costs and benefits were valued in 2020-Norwegian kroner (NOK)^1^ or included as a ‘non-monetized’ effect if monetization was not feasible (e.g., due to lack of data or an established valuation method). For publicly financed services, we added a tax financing cost of 20 percent, in line with Norwegian guidelines [25, 26].

To deliver the telemedicine-based intervention as a primary care service, infrastructure (i.e., personnel, office space, welfare technology, information technology and training of personnel) is required. In addition, GPs are required to participate in a multidisciplinary collaborative meeting when patients start using telemedicine. These costs were quantified and valued using separate registration forms completed by the local centers, supplemented with fee schedules and national wage statistics from Statistics Norway. Although the implementation of the telemedicine-based intervention will likely entail costs at the national level (i.e., for the NDH), these future costs are highly uncertain and will depend on several factors, such as the service design and technologies. We therefore opted to exclude these costs from the analysis. We also considered patients’ time and travel costs associated with receiving the intervention.

To quantify consequences for healthcare utilization, we used estimates from the effectiveness analysis comparing healthcare utilization for the intervention group with the control group. We included healthcare utilization outcomes when differences between the intervention and control group were statistically significant. The costs of these services were valued using fee schedules and accounting data from the municipalities.

We used quality-adjusted life-years (QALYs) [27] as an indicator of changes in health, and informed QALY estimates using the estimates from the effectiveness analysis of EQ-5D-5L questionnaires. In the absence of an explicit willingness-to-pay threshold for additional QALYs in Norway, we used commonly cited thresholds [28, 29] of NOK 700,000 (NOK 400,000 – NOK 1,4 million) to value QALY gains across scenarios. To evaluate the impact of telemedicine on user experience, we analyzed data from interviews and literature. To our knowledge, there is no methodological framework for quantifying and valuing consequences on user experience, although a recent Norwegian White Paper [28] suggests that effects on coping and self-efficacy may be incorporated in the QALY estimate. As such, we included effects on user experience in a descriptive manner, so-called non-monetized effects.

In a cost–benefit framework, an intervention is considered “cost-effective” if the monetized value of benefits (of the intervention compared to usual care) exceeds the additional monetized cost of the intervention, in which case the intervention provides a “net positive societal value”. In addition, we considered to what extent non-monetized effects contribute to societal value.

To reflect uncertainty, each effect was presented with three scenarios: 1) ‘most likely’, reflecting our best guess, 2) ‘optimistic’, reflecting a situation with efficient organization and patient selection, resulting in lower costs and higher benefits, and 3) ‘pessimistic’, reflecting inefficient organization and patient selection, resulting in higher costs and lower benefits. We also conducted an uncertainty analysis to assess the impact of key assumptions on the societal value of the intervention. Finally, we evaluated distributional effects to inform the distribution of benefits and costs between primary and specialist healthcare services, as well as in the general population and for GPs, municipalities, and health trusts.

### Ethics

The Regional Committee for Medical and Health Research Ethics assessed the study protocol (2018/1927). A Data Protection Impact Assessment, conducted together with the Norwegian Centre for Research Data in January 2019, concluded that the data collection and storage were conducted in accordance with the GDPR (988680). Participants received written and oral information about the trial upon inclusion and signed a consent form. Participation was voluntary, and the patient could withdraw from the trial at any time. The protocol was registered in www.clinicaltrials.gov (NCT04142710).

## Trial participants

735 patients were included in the trial, of whom 5 withdrew their consent to participate after inclusion. Of the remaining 730 participants, 261 participants were randomized into the intervention group and 276 to the control group. 193 participants were included in the non-randomized intervention group. Thus, 454 participants received telemedicine-based follow-up. For 4 patients, the project staff had registered faulty social security numbers, for which reason register data could not be obtained. These participants are thus not included in the analysis. 12 months after inclusion, 24 percent of the participants (177 patients) had ended their participation in the trial. Of these, 67 participants had died, while 110 participants discontinued their participation for other reasons, e.g., the technology was too challenging (20), deterioration of health status (14), an experience that the benefit of the intervention did not outweigh the cost (13), and other (63). Of the 110 participants who discontinued their participation, 87 participants consented to further data collection after their discontinued participation and were included in the analysis, while the remaining 23 participants were excluded from the analysis. Consequently, 636 participants were included in the study population, of which 395 were included in the intervention group and 241 in the control group (Table 1). Withdrawal was more common in the intervention group (*p* = 0.000) because there was no cost related to control group participation. Withdrawal was not differential in terms of gender, age, or diagnosis.

## Results

### Health status

#### Health-related quality of life (HRQoL)

Since data for clinical outcomes were not available for the control group, we rely on patient-reported health outcomes. Changes in HRQoL measured with EQ-5D-5L and VAS at 12 months are presented in Table 2. Intervention group participants reported a significantly more positive development in both measures than the control group in the first 12 months after inclusion. While the control group experienced a negative average change in the HRQoL value and VAS score, the average values for the intervention group remained more stable from baseline to 12 months follow-up. The intervention group did, however, experience a small decrease in VAS.

**Table 2:** HRQoL measured by EQ-5D-5L and EQ VAS questionnaire at inclusion and 12 months after inclusion

	Intervention		Control		DiD	*p*-value	*N*
	Before	After	Before	After			
Composite HRQoL (EQ-5D-5L)	0.638	0.643	0.659	0.617	0.05*	0.064	291
VAS	54.6	53.9	55.1	48.1	6.38***	0.004	286

Questionnaire responses by participants at inclusion and 12 months after inclusion. *N* refers to the number of patients who answered the patient questionnaire at inclusion and at 12 months after inclusion. UK tariffs are applied for computing HRQoL values. *p*-values are from two-sided *t*-tests: *p < 0.1, **p < 0.05, ***p < 0.01. Source: Participant questionnaire

Under the assumption that HRQoL changes linearly over time and using the individual reported HRQoL values at inclusion and 12 months after inclusion, there was a significant difference in the number of QALYs over the 12 months period, corresponding to a gain of 0.0161 QALYs from the intervention (*p*-value = 0.014, *N* = 248).

#### Survival

To ensure a similar 12-month follow-up period for all individuals when estimating 12 months of absolute risk reduction, the test sample consisted of participants who were included before April 2020 (Table 3). The absolute risk reduction was 0.04 (*p* = 0.080 with *t*-test).

**Table 3:** Comparing survival in the treatment group and control group twelve months after inclusion

	Intervention group	Control group	*p*-value from two-sided *t*-test
Percentage share of patients who died	7	11	0.080

Source: Norwegian Patient Registry (NPR)

### User experience

Twelve months after inclusion, participants in the intervention group were significantly more satisfied with the follow-up of their health compared to the control group (Table 4, first row).

**Table 4:** Perceived quality of the healthcare service, control and understanding of own illness at inclusion and 12 months after inclusion

To what extent **…**	Intervention		Control		DiD	*p*-value	*N*
	Before	After	Before	After			
… are you satisfied with the follow-up of your own health?	3.487	3.551	3.500	3.250	0.314***	0.006	244
… do you experience that you understand your body's signals and symptoms?	3.272	3.370	3.477	3.367	0.208**	0.033	282
… do you feel that you have control over your health situation?	3.012	3.088	3.093	2.963	0.207*	0.063	278

Questionnaire responses by participants at inclusion and after 12 months. The values in the table are calculated as the average of all answers within each group. Respondents could choose between the following answer options: "Not at all" (1), "To a small extent" (2), "To some extent" (3), "To a large extent" (4) and "Do not know" (−). Respondents who answered "Do not know" were excluded from the analysis. *p < 0.1, **p < 0.05, ***p < 0.01. Source: Participant questionnaire

Twelve months after inclusion, participants in the intervention group reported a significantly better development in their understanding of their body’s signals and symptoms (Table 4, second row), and better control of their health situation (row 3), compared to the control group.

### Use of healthcare services

#### Home-based care services (HCS)

Participants in the intervention group experienced an 8.2 percentage point reduction in the proportion of HCS compared with the control group 11–13 months after inclusion (Table 5). Among those who had HCS when the trial started, we found a reduction both in the number of visits (− 93, *p* = 0.059) and the number of minutes (− 1526, *p* = 0.033) in the intervention group compared with the control group in the 12-month period after inclusion. We also found a 3.5 percentage point reduction in the proportion of the intervention group that had practical assistance relative to the control group.

**Table 5:** Proportion of participants who received HCS in the intervention group compared with the control group from inclusion to 11–13 months after inclusion

	Intervention		Control		DiD	*p*-value	*N*
	Before	After	Before	After			
Home-based care services	32.7	25.3	27.9	28.7	− 8.2**	0.013	636
Practical assistance at home	15.7	14.7	12.5	15.0	− 3.5*	0.081	636

*p*-values calculated from *t*-tests: *p < 0.1, **p < 0.05, ***p < 0.01. Source: Registration by the municipalities

#### GP services

Table 6 shows an increase in the number of GP contacts in the intervention group relative to the control group. This increase was mainly driven by an increase in the number of multidisciplinary contacts between a patient’s GP and other health personnel in the municipality.

**Table 6:** Number of GP services per three-month period from 18 months before study inclusion to 12 months after inclusion

	Intervention		Control		DiD	*p*-value	N
	Before	After	Before	After			
Number of contacts with the GP	4.75	5.88	4.60	5.02	0.92**	0.000	636
Number of consultations with the GP	1.87	1.95	1.86	1.80	0.15	0.155	636
Number of multidisciplinary collaborative meetings with the GP	0.02	0.05	0.02	0.02	0.10***	0.000	636
Number of multidisciplinary contacts with the GP using phone or message	0.53	1.41	0.06	0.85	0.70***	0.000	636

Contacts cover tariffs 1ad, 1ad2, 1be, 1bd, 1bk, 1i, 1 h, and 1 g. Consultations cover tariffs 2ad, 2ae, 2aek and 2fk. Multidisciplinary collaborative meetings correspond to tariff 14 and multidisciplinary collaborative contacts with the GP using phone or message correspond to tariff 1f. *p*-values calculated from *t*-tests: *p < 0.1, **p < 0.05, ***p < 0.01. Source: Norwegian GP Registry

We did not detect differences in utilization of out-of-hours services in the intervention group compared to the control group.

#### Specialized healthcare services

While the telemedicine-based intervention could impact the use of specialized (hospital) healthcare services, no difference was detected in register data with respect to planned outpatient hospital consultations, day treatments, or in-patient care (Table 7).

**Table 7:** Number of specialized healthcare services per three-month period from 18 month before study inclusion to 12 months after inclusion

	Intervention		Control		DiD	*p*-value	*N*
	Before	After	Before	After			
Number of outpatient hospital consultations	1.65	1.86	1.76	2.03	0.20	0.243	636
Number of hospital day treatments	0.12	0.20	0.11	0.15	0.06	0.251	636
Number of planned hospital admissions	0.11	0.10	0.09	0.08	0.00	0.831	636
Number of unplanned hospital admissions	0.29	0.34	0.30	0.25	0.08	0.152	636

*p*-values calculated from *t*-tests: *p < 0.1, **p < 0.05, ***p < 0.01. Source: Norwegian Patient Registry (NPR)

### Cost–benefit analysis

#### Resource use associated with the delivery of the telemedicine-based intervention

We utilized the Norwegian framework for cost–benefit analyses. The total cost of delivering the telemedicine-based intervention for 12 months amounted to NOK 42,440 per patient in our ‘most likely’ scenario and ranged from NOK 20,710 to NOK 91,010 in our ‘optimistic’ and ‘pessimistic’ scenarios, respectively.^2^ The costs for the primary care service were the main cost driver and amounted to NOK 35,370 in our most likely scenario. The patients’ time costs were negligible as most participants reported that they only spent a few minutes per day to administer measurements and follow-up. Thus, we only included a time cost of four hours per month in our ‘pessimistic’ scenario.

#### Consequences of the telemedicine-based intervention

The telemedicine-based intervention contributed to 1) a reduction of 25 h of home-based care services for the patients who received these services at enrollment (one-third of participants), 2) an increase of 0.4 multidisciplinary collaborative meetings with the GP and 3) three additional multidisciplinary contacts with the GP using phone or message. In sum, the telemedicine-based intervention contributed to net savings of healthcare of NOK 6530 per patient per year (NOK 7840 – NOK 5220). The telemedicine intervention contributed to a QALY gain of 0,0161 QALYs, which given a willingness-to-pay threshold of NOK 700,000 corresponds to a net societal value of NOK 11,270 (NOK 6440 – NOK 22,540) per patient who received the telemedicine-based intervention for a 12-month period. The most important effects of the intervention on user experience were increased feelings of safety and self-efficacy, improved satisfaction with the follow-up from the healthcare service, as well as a minor improvement in user involvement and next-of-kin experience.

#### Net societal value

In sum, in our ‘most likely’ scenario, the societal value of the intervention on health, and healthcare utilization, did not outweigh the costs associated with the delivery of the service, resulting in a net societal loss of NOK 24,640 per patient receiving follow-up for 12 months (Table 8). Consequently, when only considering monetized consequences, the telemedicine-based intervention would not be considered cost-effective. However, the intervention improved user experience and self-efficacy, which represent societal value. This implies that the intervention would only be considered cost-effective if decision-makers' willingness-to-pay for improved user experience and self-efficacy is at least NOK 24,640 per patient per year.

**Table 8:** The societal value of the intervention, per patient with 12 months of follow-up

Consequence for society	‘Most likely’ scenario (2020-NOK)	‘Optimistic’ scenario (2020-NOK)	‘Pessimistic scenario (2020-NOK)
Resource use associated with the delivery of the intervention Costs for the primary care sector Costs for the user (pessimistic scenario) Tax financing	− 42,440	− 20,710	− 91,010
Improved physical and mental health (societal benefit) Health-related quality of life	+ 11,270	+ 22,540	+ 6440
Changes in healthcare utilization Costs for the healthcare service Tax financing	+ 6530	+ 7840	+ 5220
Net societal value (sum of monetized consequences)	− **24,640**	** + 9670**	− **79,350**
Non-monetized consequence: Improved user experience Safety and self-efficacy Satisfaction with follow-up User involvement Next-of-kin experience			

Plus indicates a societal benefit, minus indicates a societal cost

In our ‘optimistic scenario’, when we assumed a higher value of improvements in health-related quality of life, accompanied by a lower cost associated with service delivery, the telemedicine-based intervention had a positive net societal value of NOK 9670 per patient. In contrast, in our ‘pessimistic scenario’, when assuming a higher cost of service delivery combined with a lower value of health-related quality-of-life improvements, the telemedicine-based intervention implied a net societal loss of NOK 79,350 per patient. In this 'pessimistic scenario’, a more-than-threefold increase in decision-makers willingness to pay for improved user experience and self-efficacy is required for the telemedicine-based intervention to be considered cost-effective, compared to our ‘most likely’ scenario.

#### Distributional effects and equity

The telemedicine-based intervention incurs costs to the municipalities, and we expected cost savings from less use of specialist healthcare services, but this was not the case in the trial. The telemedicine-based intervention may not be equitable in the sense that it excludes patients with a lack of technology competence.

## Discussion

The purpose of this trial was to compare the outcomes of a complex telemedicine-based intervention that provides primary healthcare to patients with chronic conditions to usual care. We show that the telemedicine-based intervention contributes to increased satisfaction with follow-up of the participants’ health, and to increased security and self-efficacy compared to usual care. These findings are in line with previous research [4]. Telemedicine-based intervention participants also avoided a deterioration in their health in the first year after inclusion, which is reflected in a significant QALY gain that is comparable to that of other similar services [30]. Telemedicine users reduced their use of home-based care services more than the control group, but we did not observe significant changes in the use of specialist healthcare services. The use of GP services increased in the intervention group, mainly because of an increase in communication between the GP and other healthcare providers about patients.

Our cost–benefit analysis shows that the costs of providing the intervention likely exceed the benefits that can be monetized. However, the intervention contributes to non-monetized effects such as safety and self-efficacy, which are outcomes decision-makers may be willing to pay for. We find that monetized benefits may outweigh the costs if the service is targeted at patients with the largest expected benefits while ensuring cost containment.

We knew from the start that evaluating the trial would be challenging for many reasons. The intervention is complex both with respect to the heterogeneity in the target group, the design and implementation of the intervention, and how the intervention interacts with other healthcare providers. In addition, the NDH wanted a study design that could quantify effects on user experience, health status and use of healthcare services, and they expected service development in the trial period, meaning that the intervention underwent continuous revision. To our knowledge, there is no study design that can easily account for all these aspects.

After thorough consideration of different study designs alongside the needs and prerequisites of the NDH and other practical matters, the trial was designed as a pragmatic randomized controlled trial and built on comprehensive data collection through registries, surveys, and interviews. One important motivation for choosing a randomized design was the need for a comparable control group. A previous trial of the same intervention also run by the NDH [31], concluded that it was impossible to draw conclusions about causal effects on e.g., use of healthcare services without a control group. Using a randomized control design, we obtained a comparable control group that provided important information about the counterfactual development of the intervention group, thus allowing us to perform quantitative analyses of the outcome measures. Further, the mixed methods approach provided qualitative insights of implementation and mechanisms of impact. The qualitative and quantitative data were further combined in a cost–benefit analysis, which informs policy implementation as cost-efficiency is an explicit priority setting criteria for healthcare interventions.

There are, however, issues related to the study design. The continuous service development and evolving technology indicate that we are evaluating a moving target. This is challenging in a randomized trial, where the treatment in both the intervention and control group usually is fixed. The service development and the large degree of heterogeneity in the intervention, usual care, and the study population mean that the quantitative analysis of our study is only informative of the effect of the intervention compared to usual care averaged over different variations of the service and patient groups. We advocate that this is useful knowledge, even if it is difficult to know *what* brings about the effects and for *whom,* especially since this is how the service is intended to be implemented in practice. While planning the trial, limiting the inclusion criteria to certain diagnoses to reduce heterogeneity and increase the precision of the quantitative analyses was discussed. Uncertainty about the size of the recruitment pool in the participating municipalities, among other things, talked against this. However, one of the most important strengths revealed from the process evaluation was that the service is not diagnosis-specific, but rather has a more holistic approach to the patients and their needs [19]. Another strength of the service turned out to be its large degree of flexibility to adapt to different patient groups and organizations.

Despite the design challenges, the study provides important knowledge about this telemedicine-based intervention. In addition, the process evaluation shows how the intervention was implemented and points to important prerequisites for success. These insights may be useful also for other interventions in different settings.

In recent years, there has been an increasing interest in telemedicine-based services, and telemedicine is often mentioned as an important solution to the challenges the healthcare sector faces in terms of aging populations and lack of health personnel. Still, empirical studies of telemedicine-based solutions struggle to identify statistically significant reductions in resource use, improved patient outcomes, and advances in health equity which has resulted in skepticism among researchers and practitioners [32].

Furthermore, previous studies have stated that it is challenging to generalize findings from research in this field due to the complexity of the interventions and the heterogeneity of the target groups [14]. This also applies to our trial in that the results may be different with another design of the intervention, other patient groups, a longer follow-up period, or when healthcare professionals have become better acquainted with the intervention. Another challenge that is common to our and previous studies, is that the service is implemented in a complex healthcare system with many stakeholders, established areas of responsibility, and ways of conduct. For the intervention to reach full effect, it is important for it to be fully integrated into the surrounding systems. This may require changes also in the surrounding structures. An insight from the evaluation can serve as an example of this. A prerequisite for reducing the number of GP consultations is that the nurses at the follow-up center feel confident in their role and have the expertise to make independent decisions based on the information they receive about users through digital home monitoring. Acquiring this confidence and competence takes time, but it might also be necessary to implement other measures to facilitate the development. Increased emphasis on digital monitoring and remote follow-up in nursing education might be a move forward.

Our process evaluation showed that it takes time and dedicated resources to build a new service, to raise awareness, and to integrate it into the existing healthcare system [19], and this is something that future research and real-life implementation should take into consideration. During a limited trial period, there may be dedicated project personnel that work to promote the new service, which may speed up the assimilation. In real-life implementation, on the other hand, such resources may be limited, which again may hamper effective implementation and integration of services, thus lowering its value. The process evaluation also showed that health personnel may be reluctant to start using new services, both because of time constraints and because of uncertainty of costs and benefits for themselves and their patients. This may impede the implementation and highlight the need for long enough follow-up in the evaluation of new services.

Despite the challenges, this study demonstrates that telemedicine-based interventions have the potential to become an important part of future primary healthcare *if organized effectively*. Implementation in clinical practice likely requires a trial-and-error approach, as the field is still developing and there are vast possibilities to adapt the service to different organizations and patient groups. Such implementation should be accompanied by research-based evaluations of both effects and costs to inform the design of future telemedicine-based interventions, and evaluations should be seen in relation to each other to reach a broader understanding of what works and what does not seem to work. Further, evaluations should preferably be based on mixed-methods approaches, which includes the possibility of comparing intervention and control groups, to reach a better understanding of the results and complexity of telemedicine-based interventions.

## Appendix: Additional material

(See Tables

**Table 9:** Data collection

Data source or method	Type of information	Timepoint
Patient questionnaire	Health related quality of life (EQ-5D-5L), valuation of own physical and mental health, coping, user experience, demographics	Enrollment, 6, 12 and 18 months after enrollment
Registration form/municipalities' electronic health record systems	Background information, participation	Enrollment
Norwegian GP registry, Norwegian Registry for Primary Health Care (KPR), Norwegian Patient Registry (NPR), Norwegian Prescription Database	GP consultations, home-based care visits, emergency unit contacts, outpatient consultations and day treatments, number of hospital admissions, drug use	2017–2021
Interviews with patients (*N*_2019_ = 23, *N*_2021_ = 25) and next of kin (N_2019_ = 11, *N*_2021_ = 8)	Patient experience, sense of security, coping, health status, time-use, shift of responsibility and tasks	2019, 2021
Interviews with follow-up center administration (*N*_2019_ = 13, *N*_2020_ = 12, *N*_2021_ = 10) and nurses (*N*_2019_ = 11), GPs (*N*_2019_ = 10, *N*_202_ = 6), and hospital and primary healthcare staff (*N*_2020_ = 4, *N*_2021_ = 11))	Recruitment, inclusion and exclusion criteria, organization, follow-up of patients, collaboration with other healthcare providers, costs and benefits, success criteria and challenges for telemedicine follow-up	2018, 2019, 2020, 2021
GP questionnaire (*N* = 39)		2020
Follow-up center nurse questionnaire (*N*_2019_ = 31, *N*_2020_ = 44)		2019, 2020

N with subscript indicates the number of informants interviewed in each year

9 and

**Table 10:** Robustness test of results comparing intervention groups and study populations

Intervention group	Randomized and non-randomized			Randomized		
Study population	PP	ITT 1	ITT 2	PP	ITT 1	ITT 2
*A. Municipal home-based care services*						
Home-based care services	− 8.2**	− 9.0***	− 9.7***	− 2.1	− 2.5	− 3.1
Practical assistance at home	− 3.5*	− 3.3	− 3.5*	− 1.2	− 1.1	− 1.5
N	635	702	725	470	521	535
*B. GP services*						
Number of contacts with the GP	0.92***	0.86***	0.82***	0.92***	0.83***	0.79***
Number of consultations with the GP	0.15	0.16	0.16	0.04	0.07	0.07
Number of multidisciplinary collaborative meetings with the GP	0.10***	0.09***	0.09***	0.10***	0.10***	0.09***
Number of multidisciplinary contacts with the GP using phone or message	0.70***	0.65***	0.62***	0.68***	0.62***	0.59***
N	636	703	726	471	522	536
*C. Specialized healthcare services*						
Number of outpatient hospital consultations	0.20	0.18	0.17	− 0.05	− 0.04	− 0.04
Number of hospital day treatments	0.06	0.02	0.02	0.09	0.07	0.07
Number of planned hospital admissions	0.00	0.01	0.01	0.00	0.01	0.01
Number of unplanned hospital admissions	0.08	0.07	0.07	0.08	0.07	0.07
N	636	703	726	471	522	536

In the first three columns, the intervention group consists of randomized and non-randomized participants. In the last three columns, non-randomized participants are excluded. The PP (per protocol) sample consists of participants who were still in follow-up 12 months after inclusion. ITT (intention-to-treat) 1 includes the PP population and participants who died within 12 months of inclusion. ITT2 includes the ITT1 population and participants who discontinued follow-up due to other reasons within 12 months of inclusion. Contacts with the GP cover tariffs 1ad, 1ad2, 1be, 1bd, 1bk, 1i, 1 h, and 1 g. Consultations with the GP cover tariffs 2ad, 2ae, 2aek and 2fk. Multidisciplinary collaborative meetings with the GP correspond to tariff 14 and multidisciplinary collaborative contacts with the GP using phone or message correspond to tariff 1f. *p*-values calculated from *t*-tests: *p < 0.1, **p < 0.05, ***p < 0.01. Source: Registration by the municipalities, Norwegian GP Registry, Norwegian Patient Registry (NPR)

10).

## Footnotes

1 In 2020, NOK 100 corresponded to EUR 9.33. Norges Bank. Exchange rates. 2022; Available from: https://www.norges-bank.no/en/topics/Statistics/exchange_rates.

2 In 2020, NOK 100 corresponded to EUR 9.33. Norges Bank. Exchange rates. 2022; Available from: https://www.norges-bank.no/en/topics/Statistics/exchange_rates.
